# Melatonin Induced Cold Tolerance in Plants: Physiological and Molecular Responses

**DOI:** 10.3389/fpls.2022.843071

**Published:** 2022-03-14

**Authors:** Sameer H. Qari, Muhammad Umair Hassan, Muhammad Umer Chattha, Athar Mahmood, Maria Naqve, Muhammad Nawaz, Lorenzo Barbanti, Maryam A. Alahdal, Maha Aljabri

**Affiliations:** ^1^Department of Biology, Al-Jumum University College, Umm Al-Qura University, Makkah, Saudi Arabia; ^2^Research Center on Ecological Sciences, Jiangxi Agricultural University, Nanchang, China; ^3^Department of Agronomy, University of Agriculture, Faisalabad, Pakistan; ^4^Department of Botany, University of Agriculture, Faisalabad, Pakistan; ^5^Department of Agricultural Engineering, Khwaja Fareed University of Engineering and Information Technology, Rahim Yar Khan, Pakistan; ^6^Department of Agricultural and Food Sciences, University of Bologna, Bologna, Italy; ^7^Department of Biology, Faculty of Applied Sciences, Umm Al-Qura University, Makkah, Saudi Arabia; ^8^Department of Biology, Research Laboratories Centre, Faculty of Applied Science, Umm Al-Qura University, Makkah, Saudi Arabia

**Keywords:** antioxidants, cold stress, gene expression, melatonin, oxidative stress, photosynthesis

## Abstract

Cold stress is one of the most limiting factors for plant growth and development. Cold stress adversely affects plant physiology, molecular and biochemical processes by determining oxidative stress, poor nutrient and water uptake, disorganization of cellular membranes and reduced photosynthetic efficiency. Therefore, to recover impaired plant functions under cold stress, the application of bio-stimulants can be considered a suitable approach. Melatonin (MT) is a critical bio-stimulant that has often shown to enhance plant performance under cold stress. Melatonin application improved plant growth and tolerance to cold stress by maintaining membrane integrity, plant water content, stomatal opening, photosynthetic efficiency, nutrient and water uptake, redox homeostasis, accumulation of osmolytes, hormones and secondary metabolites, and the scavenging of reactive oxygen species (ROS) through improved antioxidant activities and increase in expression of stress-responsive genes. Thus, it is essential to understand the mechanisms of MT induced cold tolerance and identify the diverse research gaps necessitating to be addressed in future research programs. This review discusses MT involvement in the control of various physiological and molecular responses for inducing cold tolerance. We also shed light on engineering MT biosynthesis for improving the cold tolerance in plants. Moreover, we highlighted areas where future research is needed to make MT a vital antioxidant conferring cold tolerance to plants.

## Introduction

Cold stress is a severe abiotic stress that significantly limits crop growth and productivity, particularly in temperate areas (Aazami et al., 2021; Feng et al., 2021). Cold stress induces severe alterations in plant physiological, biochemical, metabolic and molecular processes, resulting in a significant reduction in crop productivity (Hu et al., 2016; Repkina et al., 2021). The plasma membrane is considered the first place affected by cold stress (Barrero et al., 2017). Exposure to cold stress substantially alters lipid composition and increases fatty acid saturation (Lado et al., 2016). Low temperature reduces water uptake, and inadequate moisture in aboveground organs leads to drought stress (Aroca et al., 2012; Hussain et al., 2018). In turn, the onset of drought owing to cold stress causes a significant reduction in root growth, nutrient, and water uptake (Nezhadahmadi et al., 2013; Hassan et al., 2017). Low temperature also induces the production of reactive oxygen species (ROS) (Hu et al., 2016; Dey et al., 2021) that damage the proteins, lipids and resultantly inhibit plant growth and, eventually, its productivity (Hassan et al., 2019, 2020, 2021). However, plants have an excellent antioxidant system to cope with ROS under stress conditions (Chattha et al., 2021; Dustgeer et al., 2021; Imran et al., 2021; Seleiman et al., 2021; Sultan et al., 2021). Additionally, plants can neutralize the impact of cold stress by accumulating various osmolytes such as proline, glycine betaine, soluble sugars, and proteins (Erdal, 2012; Ghosh et al., 2021). However, the accumulation of these osmolytes varies depending on crop species and stress conditions (Erdal, 2012). The accumulation of these osmolytes protects the membranes and ensures better growth and production in cold stress (Sun et al., 2020; Ghosh et al., 2021).

Melatonin (*N*-acetyl-5-methoxytryptamine) (MT) is an imperious endogenous molecule that possesses excellent antioxidant properties (Arnao and Hernández-Ruiz, 2015a; Kołodziejczyk et al., 2021). MT is involved in different processes ranging from root growth (Zhang et al., 2014), flower development, fruit ripening, photosynthesis (Tan et al., 2012), leaf senescence (Byeon et al., 2012; Wang et al., 2013), and alleviation of stress-induced oxidative damage (Wang et al., 2014; Arnao and Hernández-Ruiz, 2015b; Shi et al., 2015a; Kołodziejczyk et al., 2021). The application of MT improves antioxidant activities performing ROS scavenging and conferring cold tolerance to plants (Zhang H. et al., 2021). Melatonin improves gene expression, which regulates the antioxidant activities and redox status under cold stress (Wang et al., 2017; Li et al., 2018a). MT reduces cold-induced inhibition in photosynthesis and photosystem-II (PS-II) activities by increasing antioxidant activities (Han et al., 2017). Moreover, MT also improves the cold tolerance by degradation of starch and increasing the electron transport and antioxidant activities (Li et al., 2018b). In recent years, many functions of MT have been identified, among which contributing to stress tolerance. Therefore, in this review we systematically discussed the potential regulatory mechanism of MT to induce cold tolerance. Further, we also focused on the future directions to make MT an essential antioxidant for cold tolerance.

## Melatonin Biosynthesis in Plants

**FIGURE 1:**
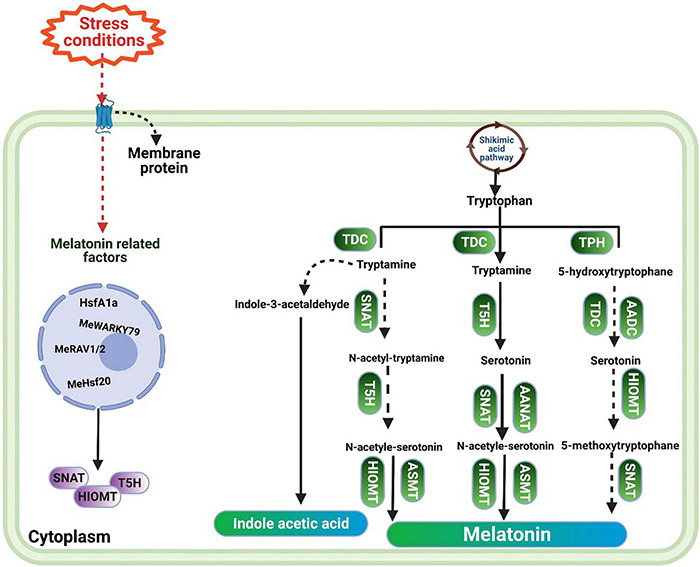
The pathway of melatonin biosynthesis in plants.

Tryptophan (TP) is considered a precursor of MT. The conversion of TP into MT involves four enzymatic reactions (Figure 1). The first step consists of converting TP into tryptamine by tryptophan decarboxylase (TDC). Tryptamine is converted into serotonin (ST) by the action of an enzyme named tryptamine 5-hydroxylase (T5H) (Posmyk and Janas, 2009). Later on, ST is converted into *N*-acetyl-serotonin by means of *N*-acetyltransferase (SNAT) or arylalkylamine *N*-acetyltransferase (AANAT). Afterward, *N*-acetyl-serotonin is converted into MT by *N*-acetyl-serotonin methyltransferase (ASMT) or hydroxyindole-O-methyltransferase (HIOMT) (Zuo et al., 2014). In parallel to this, ST is also converted by HIOMT into the 5-methoxytryptamine, which in turn is converted by SNAT into the final product, MT (Tan et al., 2016). A recent study also identified the reverse pathway for MT biosynthesis, in which *N*-acetyl-serotonin deacetylase catalyzes *N*-acetyl-serotonin into serotonin (Lee et al., 2017). As a precursor of MT, tryptophan is also a precursor of indole-3-acetic-acid (IAA). Tryptamine pathway is one of the pathways of IAA synthesis, in which TP is converted into tryptamine, and then tryptamine is converted into IAA by indole-3-acetaldehyde (Wang J. et al., 2012; Wang Y. Y. et al., 2012). This similarity explains why MT has effects similar to those of IAA, as it has been reported that MT improves vegetative growth to an extent comparable with IAA (Hernandez-Ruiz et al., 2004).

## Endogenous Melatonin Biosynthesis in Plants

Plant chloroplast and mitochondria are considered important sites of MT biosynthesis (Tan et al., 2013). MT biosynthesis has been reported in many plants, including fruit trees, herbs, and crops (Byeon et al., 2012). The levels of MT synthesis in plants are subjected to seasons and circadian rhythms (Beilby et al., 2015). Additionally, MT concentrations vary among plant species, organs, and growth stages (Hernández-Ruiz and Arnao, 2008). For instance, in morning glory, the MT concentration was significantly increased during the maturation period (Van-Tassel et al., 2001). Lastly, environmental conditions significantly affect MT synthesis in plants; for instance, MT concentration was significantly higher in field grown rice compared to the growth chamber (Byeon et al., 2012). Similarly, MT levels were also significantly higher in grapevine plants grown under illumination than under darkness, indicating that light signals induce MT synthesis (Boccalandro et al., 2011). In contrast to this, another source reports that MT synthesis in grapevine was significantly higher during the night compared to the day, which indicates that light inhibits the MT biosynthesis in these species (Arnao and Hernández-Ruiz, 2013b).

## Abiotic Stress Induced Melatonin Biosynthesis in Plants

Melatonin, an excellent antioxidant, interacts with ROS and reduces ROS production and its damaging effects under stress conditions (Arnao and Hernández-Ruiz, 2013a). Therefore, in stress conditions, the increases in MT synthesis is linked with an increase in ROS (Arnao and Hernández-Ruiz, 2013b). The concentration of MT in grapevine and barley was significantly increased in stress conditions, and the level of MT was further enhanced by increasing the stress intensity (Arnao and Hernández-Ruiz, 2009). Moreover, MT synthesis in rice seedlings was also significantly increased under heat stress (Byeon and Back, 2013). MT biosynthesis considerately increased on exposure to stress, proving that MT plays an imperative role in plants’ response to different stresses (Hardeland, 2016).

Melatonin biosynthesis in plants is related to gene expression and enzymatic activities responsible for MT biosynthesis. For instance, an increase in genes expression (TDC: tryptophan decarboxylase: TDC, T5H: tyrosine gene) significantly increased MT synthesis in rice seedlings grown under cadmium stress (Byeon et al., 2015). Moreover, an increase in MT in rice was also linked with SNAT and ASMT under high temperatures (Byeon and Back, 2013). Generally, the concentration of MT in plants is strongly correlated with the availability of its precursors (Byeon et al., 2015), and ST plays a crucial role to improve cold tolerance (Kang et al., 2010). Moreover, a higher level of 2-hydroxymelatonin under cold and drought stress in rice indicates its role in plant resistance to these stresses (Lee and Back, 2016). Additionally, in the tomato crop, the concentration of MT was significantly increased by direct binding of a transcription factor (HsfA1a) to the caffeic acid O-methyltransferase 1 (COMT1) gene promoter under Cd stress (Cai et al., 2017).

## Effect of Cold Stress on Plants

**FIGURE 2:**
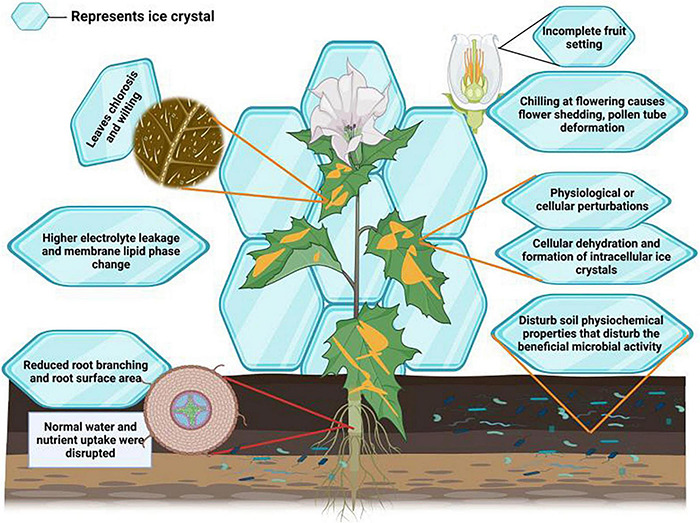
Effect of cold stress on plants. Cold stress induces the formation of crystal which reduces membrane integrity, causes electrolyte leakage and lipid saturation, reduces root growth which in turn decreases the water and nutrient uptake. Moreover, cold stress also causes leaf wilting and chlorosis and disturbs photosynthetic performance and microbial activities, and induces flowering shedding, deformation of pollen tube, incomplete fruit setting and results in significant growth and yield losses.

Cold stress induces several morphological alterations in plants and causes a reduction in growth and productivity (Equiza et al., 2001). Cold stress determines leaf chlorosis and wilting, leading to necrosis and stunted growth (Janowiak et al., 2002). Cold stress delayed and reduced wheat germination, reducing stand establishment and final productivity (Jame and Cutforth, 2004). Cold stress limits root proliferation, growth and surface areas (Figure 2), leading to a substantial reduction in nutrient and water uptake (Hussain et al., 2018; Kul et al., 2020). The reproductive stage of plant life is also susceptible to cold stress (Thakur et al., 2010). For instance, cold stress causes shedding of flowers, deforms pollen tubes (Chakrabarti et al., 2011), induces pollen sterility (Ji et al., 2017), and disrupts grain development (Barton et al., 2014), consequently causing a reduction in final productivity (Hussain et al., 2018).

Cold stress severely alters plant physiological processes. Plants need to maintain membrane stability in stress conditions; however, cold stress reduces membrane stability (Table 1) and protein structures (Chen et al., 2018). Cold stress induces the formation of ice crystals in plant tissues (Puhakainen, 2004), which reduces apoplastic water potential and results in the flow of water from cells. Thus, cold stress at the cellular level, often followed by drought stress, seriously reduces growth and productivity (Hassan et al., 2021). This onset of drought stress reduces root growth (Table 1), root hydraulic conductivity and turgidity of plant leaves (Siddique et al., 2000). Resultantly, plant water and nutrient uptake and carbohydrate metabolism are seriously disrupted, involving significant yield losses (Hassan et al., 2021). Besides this, lower temperature also disturbs soil microbial activities, affecting plant nutrient relationships (Massenssini et al., 2015). Ice crystals’ formation also increases electrolyte leakage and causes lipid peroxidation (Hassan et al., 2021). Ice crystals can also puncture the cells, resulting in cytosol outflow and causing plant death (Zhang F. et al., 2011; Demidchik et al., 2014; Sun et al., 2019). Thus, preventing the formation of ice crystals is considered an essential cold tolerance mechanism in plants. Plants accumulate various cryoprotective polypeptides (e.g., COR15a) and osmolytes to cope with the damaging effects of cold stress (Ritonga and Chen, 2020).

**TABLE 1:** Effect of cold stress on growth, physiological attributes, and anti-oxidant activities.

Crop	Stress conditions	Effects	References
Soybean	17/13°C DNT	Cold stress reduced the plant height, nodes production, stem biomass, pods production, biomass, and seed production.	Staniak et al., 2021
Maize	4°C	Chilling stress reduced the seedling growth, biomass production, RWC, and increased the MDA contents membrane permeability, proline accumulation, and APX, CAT, POD, and SOD activities.	Zhang Q. et al., 2021
Wheat	6°C	Cold stress reduced the root and shoot growth and biomass production, and increased the MDA and H_2_O_2_ accumulation, proline, glycine betaine accumulation, and EL.	Golizadeh and Kumleh, 2019
Stevia	5°C	Cold stress reduced efficiency of PS-II, chlorophyll contents, photosynthetic rate, and water use efficiency.	Hajihashemi et al., 2018
Chickpea	4°C	Cold stress increased EL, MDA, and H_2_O_2_ accumulation. However, cold stress also resulted in increase in activities of APX, CAT, and SOD.	Karami et al., 2018
Sunflower	−3°C	Cold stress increased the EL, reduced the chlorophyll fluorescence, osmotic potential of sunflower plants.	Helena et al., 2017
Sugarcane	4°C	Cold stress reduced the root growth, root biomass, root vigor, activities POD and SOD, MDA, proline, and soluble sugars accumulation.	Sun et al., 2017
Barley	−8°C	The cold stress increased the lipid per-oxidation, MDA and H_2_O_2_ accumulation, CAP and POD activities and decreased the membrane stability.	Valizadeh et al., 2018
Wheat	4°C	Cold stress reduced the leaf moisture contents, RWC, dry matter contents, photosynthetic, and transpiration rates of wheat crop.	Bibi et al., 2017

*DNT, day/night temperature.*

Photosynthesis is a major source of grain production, and this process is seriously affected by cold stress (Rinalducci et al., 2011; Khan et al., 2017). Cold stress causes the over-excitation of PS-II, which increases the energy loss by non-radiative reactions (Venzhik et al., 2011; Cvetkovic et al., 2017). Further, cold stress reduces chlorophyll synthesis, photosynthetic efficiency, Rubisco activity, electron transport, stomatal conductivity, which reduce the assimilates production and cause severe yield losses (Bota et al., 2004; Yamori et al., 2009; Hussain et al., 2018). Moreover, cold stress also damages mitochondria’s structure, disturbs enzymatic activities, and slows down the flow of kinetic energy, consequently diminishing the respiration rate (Ikkonen et al., 2020).

Reactive oxygen species increase under cold stress improved the oxygenation response in plant chloroplast and increased glycolate accumulation. This glycolate is converted to glyoxylate in plant peroxisomes, accompanied by accumulation of hydrogen peroxide (H_2_O_2_) (Hassan et al., 2021). However, plants have an excellent antioxidant defense system to scavenge these ROS (Ritonga and Chen, 2020). The response of plants to cold stress consists of different steps, including dictation of stress followed by signal perception, transduction, and increased expression of stress-responsive genes (Ganeshan et al., 2008). Many genes have been identified in plants that initiate a cascade of transcriptional, biochemical, and physiological processes crucial to chilling tolerance (Kosová et al., 2008). Moreover, plants accumulate various osmolytes, reduce water content, scavenge ROS and maintain carbon metabolism to counter the effects of cold stress (Ruelland and Zachowski, 2010; Thakur and Nayyar, 2013; Hassan et al., 2021). Plants also accumulate various soluble sugars that stabilize the cellular membrane on exposure to cold stress (Yokota et al., 2015). Moreover, the accumulation of osmolytes and sugars also decrease the ROS and malondialdehyde (MDA) contents under cold stress by improving catalase (CAT), peroxidase (POD), and superoxide dismutase (SOD) activities (Sun et al., 2019).

## Melatonin Improves Growth and Yield Under Cold Stress

Cold stress is a severe abiotic stress that substantially limits crop productivity by imposing serious alterations in plant physiological and metabolic processes, and hormonal imbalance, nutritional disorders, poor photosynthetic efficiency, and production of ROS (Turk and Genisel, 2020). MT is an major signaling molecule that promotes plant growth (Table 2) and development, and protects against abiotic stresses (Posmyk and Janas, 2009). Low temperature inhibits plant growth and development, in response to which, MT possesses excellent potential to counter cold influence (Table 2; Bajwa et al., 2014). Cold stress induces reduction in photosynthetic pigments; however, MT application (100 μM) significantly increases the synthesis of photosynthetic pigments, and therefore maintains plant growth under cold stress (Han et al., 2017). Cold stress induces a marked increase in MDA accumulation, lipid peroxidation and electrolyte leakage (Hulya et al., 2014; Hu et al., 2016). However, MT supplementation was shown to markedly reduce MDA accumulation and ROS deleterious impact on cellular membranes of rice seedlings, which in turn resulted in appreciably improved plant growth under cold stress (Han et al., 2017).

**TABLE 2:** Effect of melatonin application on growth and physiological and molecular attributes under cold stress.

Crop	Cold stress	MT application	Effects	References
Barley	5°C	1 μM	MT supplementation increases the germination, seedling growth, endogenous MT concentration, chlorophyll and caroteniod contents, proline and soluble proteins accumulation, and expression of HvCCA1 and HvTOC1 genes.	Chang T. et al., 2021
Watermelon	4°C	150 μM	The application of MT improves the endogenous MT contents and accumulation of MeJA, chlorophyll fluoresces, expression of ClCBF1 and ClCBF2 genes.	Li et al., 2021
Pepper	25/20°C DNT	5 μM	MT foliar spray improves the leaf area, photosynthetic rate, stomatal conductance, biomass production, water potential, proline contents, and fruit yield.	Korkmaz et al., 2021
Pistachio	25/20°C DNT	0.5 μM	MT supplementation improves the growth, chlorophyll and caroteniod and phenolic contents, carbohydrate, proline, and GABA accumulation.	Barand et al., 2020
Wheat	20°C	1 μM	MT treatment improves the plant biomass production, root/shoot ratio, nitrogen uptake and activities of nitrate reductase and glutamine synthetase.	Qiao et al., 2019
Wheat	10/4°C DNT	1 mM	MT application improves the stomatal conductance, photosynthetic efficiency and expression of Cu/Zn SOD to confer cold tolerance.	Sun et al., 2018
Tea	25/20°C DNT	500 μM	MT foliar spray increases the photosynthetic rate, efficiency of PS-II, chlorophyll contents and expression of stress proteins.	Li et al., 2018a
Bermudagrass	4°C	100 μM	MT supplementation increases the chlorophyll fluoresce and endogenous MT contents to confer the cold tolerance.	Hu et al., 2016
Bermudagrass	4°C	100 μM	MT treatment increases the chlorophyll contents, chlorophyll fluoresce, endogenous MT contents.	Fan et al., 2015
Maize	27/25°C DNT	1 mM	MT application improves the growth, chlorophyll contents, RWC and increased the concentration of Fe, Mg, K, S, B, and Zn.	Turk and Erdal, 2015

*DNT, day/night temperature.*

In the same experiment (Han et al., 2017), MT reduced the cold-induced inhibition in plant photosynthetic activities, and protected the photosynthetic apparatus by improving the antioxidant activities; all this determined better plant performance under cold stress. However, MT mediated improvement in plant growth largely depends on methods and rate of MT application under cold stress (Han et al., 2017). MT supplementation maintains higher Fv/Fm and plant water relationships, while it reduces MDA and H_2_O_2_ by improving antioxidant activities (Table 2: ascorbate peroxidase: APX, CAT, POD, and SOD), enhancing plants tolerance to cold stress (Li et al., 2018a). MT supplementation also protects the photosynthetic machinery, maintains the redox homeostasis, and enhances gene expression so as to mitigate the deleterious impacts of cold stress and improve plant growth (Li et al., 2018a). Exogenous application of MT improved plant defense to counter the harmful effects of cold stress in Bermudagrass (Fan et al., 2015). MT application also improved osmolyte accumulation, nutrient and water uptake, hormonal accumulation and enzymatic activities, which countered the effects of cold stress by strengthening the anti-oxidant defense system and improving plant growth (Irshad et al., 2021). In addition, MT supplementation also improved carbon assimilation, osmotic potential, enhanced plant water content and photosynthetic efficiency, resulting in substantial growth improvement and unconstrained development under freezing temperature (Irshad et al., 2021).

## Melatonin Maintains Membrane Stability and Improves Plant Water Relations Under Cold Stress

Membrane stability is a major damage in plants caused due to cold stress. Cold stress decreases membrane fluidity and changes the balance between transpiration and water uptake, and cause water dehydration in plant shoots (Turk et al., 2014). Eventually, it also affects the stomata movements and substantially decreases the photosynthetic rate (Hassan et al., 2021). However, MT application protects membranes and improves membrane stability by scavenging ROS through enhanced antioxidant activities (Turk et al., 2014). The foliar and seed priming with MT appreciably improved the membrane stability linked with reduced MDA and H_2_O_2_ (Table 3) accumulation (Sun et al., 2018). The increase in membrane stability reduced the EL and loss of osmolytes and conferred the cold tolerance in plants (Sun et al., 2018). Additionally, exogenous MT also improved the enzymatic and non-enzymatic antioxidant activities, maintaining the membrane integrity and conferring cold tolerance with corresponding lower electrolyte leakage (EL), MDA, and H_2_O_2_ accumulation (Table 3; Fan et al., 2015). The regulation of plant water relationships is plants are linked with plants adaptation to cold stress (Turk et al., 2014). Cold stress significantly decreased the plant relative water contents (RWC); however, MT application reduces the negative impacts of cold stress and maintains higher RWC (Pu et al., 2021). The larger leaf surface area with MT treatment may be associated with improved water contents under cold stress (Turk et al., 2014). Moreover, MT also protects the plant membranes, reducing water loss and maintaining the higher RWC under cold stress (Turk et al., 2014).

**TABLE 3:** Effect of melatonin supplementation on different oxidative stress markers under cold stress.

Crop	Cold stress	MT application	Effects	References
Common sage	20/15°C DNT	200 μM	MT application significantly reduced the MDA and H_2_O_2_ accumulation under cold stress.	Bidabadi et al., 2020
Sapota fruit	8°C	90 μM	MT application decreased electrolyte leakage, MDA contents, and production of H_2_O_2_, and O^2–^.	Mirshekari et al., 2020
Tea	4°C	100 μM	MT application reduced the H_2_O_2_, and O^2–^ and MDA accumulation under cold stress.	Li et al., 2019
Peach	4°C	100 μM	MT foliar spray reduced the MDA accumulation and production of H_2_O_2_, and O^2–^.	Cao et al., 2018
Tomato	5°C	200 μM	MT supplementation reduced the chilling injury, ion leakage, MDA accumulation and H_2_O_2_, and O^2–^production.	Azadshahraki et al., 2018
Tomato	4°C	100 μM	Exogenous MT reduced the electrolyte leakage, MDA H_2_O_2_, and O^2–^ accumulation under cold stress.	Ding et al., 2017
Pepper	15°C	25 μM	MT improved membrane stability and reduced the MDA and H_2_O_2_ accumulation.	Korkmaz et al., 2017
Melon	12/6°C DNT	400 μM	MT application reduced the MDA contents and ROS production.	Zhang et al., 2017
Bermudagrass	4°C	100 μM	Foliar MT supplementation reduced the electrolyte leakage, MDA accumulation and ROS production.	Shi et al., 2015a

*DNT, day night temperature.*

## Melatonin Improves Water and Nutrient Uptake Under Cold Stress

The potential water reduction is considered the fastest effect of chilling stress. Cold stress diminishes the water influx through plants roots due to increased water viscosity and a decrease in membrane fluidity, which reduces the cell turgor pressure (Turk et al., 2014). However, MT application improves the plant water uptake under cold stress, which indicates that MT can reduce the negative impacts of cold stress (Turk et al., 2014; Hussain et al., 2018). Exogenous MT application increases the vapor pressure deficit between the plant leaf surface and atmosphere, enabling the plant roots to improve the water uptake (Pu et al., 2021).

Cold stress alters membrane structure by disturbing various physiological and biochemical properties, disturbing multiple processes, including nutrient and water uptake (Nayyar et al., 2005). Optimum nutrient uptake and transportation is necessary for plants to maintain their physiological processes and structural integrity under cold stress (Dumlupinar et al., 2011). MT application significantly improved the nutrient uptake under cold stress. Likewise, MT application causes a significant increase in calcium (Ca) uptake under cold stress, which shows that MT achieved its protective role on membranes under cold by increasing the Ca uptake. Moreover, increased Ca uptake following MT application protects the membranes and reduces electrolyte leakage and MDA accumulation under cold stress (Turk and Erdal, 2015). MT application also maintained higher uptake of potassium (K), phosphorus (P), sulfar (S), boron (B), copper (Cu), iron (Fe), magnesium (Mg), manganese (Mn), and zinc (Zn), which improved the plant performance and confer the cold tolerance (Turk and Erdal, 2015).

Cold stress also decreased the Mg uptake which in turn decreased the chlorophyll synthesis owing to fact Mg is important constituent of chlorophyll. However, MT treatment improves the Mg uptake and ensures the better chlorophyll synthesis and subsequent photosynthetic performance under cold stress (Turk and Erdal, 2015). MT application also improved the N uptake and maintained higher N contents in plant shoot under cold stress. The increase in N uptake following MT application is attributed to higher activities of nitrate reductase (NR) and glutamine synthetase (GS) and resultantly improve the plant growth and productivity (Qiao et al., 2019). Cold stress substantially reduced the NPK however, MT application improved the NPK under cold stress (Irshad et al., 2021). Cold stress reduced the N uptake by reducing the root activities (Feng et al., 2011) nonetheless, exogenous MT application upregulates nutrient uptake by increasing root activity and enzymatic activities under cold stress (Turk and Erdal, 2015; Irshad et al., 2021).

## Melatonin Improves Hormones and Osmo-Lytes Accumulation to Confer Cold Tolerance

Osmo-lytes accumulation is one of the most important mechanisms used by plants to improve the stress tolerance (Hassan et al., 2021). The formation of viscous among cells is imperious to improve the cold tolerance; however, this formation largely depends on carbohydrate contents. The application of MT improved the carbohydrate contents which in turn improve the cold tolerance in plants (Sarropoulou et al., 2012; Turk et al., 2014). Amino acids and proteins also play an imperative role in plants tolerance to cold stress. The application MT substantially increased the MT accumulation in plants which in turns improve the plant anti-oxidant performance and confer the cold tolerance (Turk et al., 2014). Melatonin application also maintained higher proline contents under cold stress that keeps cell water contents, maintain membrane stability and increases the anti-oxidant activities to confer cold stress in plants (Turk et al., 2014). The application of MT appreciably improved the synthesis of proline enzymes including the 1-pyrroline-5-carboxylate syntheses (P5CS) and ornithine aminotransferase (OAT) which in turn improve the proline synthesis under cold stress and confer the cold tolerance were (Madebo et al., 2021). Melatonin application also improved the endogenous MT, glycine betaine and soluble sugars accumulation and resulting in substantial increase in anti-oxidant activities and subsequently in cold stress (Irshad et al., 2021).

Different hormones including, auxins (IAA), abscisic acid (ABA), gibberellins (GA3), and cytokinins (CK) play a significant role in chilling tolerance (Khan et al., 2017). The response of plants to various stresses are depends on the cross talk among the hormonal signaling pathways (Verma et al., 2016). The exogenous MT supplementation improved the IAA and GA3 concentration while MT application reduced the ABA accumulation under cold stress (Pu et al., 2021). ABA induces stomata closing and reduced the photosynthetic rate under cold stress (Lata and Prasad, 2011). Cold stress significantly increases the ABA contents (Zhang et al., 2014), however, MT application markedly reduced the ABA accumulation in cold stress (Zhao et al., 2016). The reduction in ABA accumulation under cold stress is attributed to re-opening of stomata following MT application (Pu et al., 2021). Melatonin application also induced significant increase in methyl jasmonate (MeJA) that leads to an increase in H_2_O_2_ accumulation and cold tolerance (Li et al., 2021). Nitric oxide (NO) maintains cellular homeostasis under stress conditions by repairing the stress induced oxidative damages (Zhao et al., 2007; Kaya et al., 2020). The increase in NO following MT initiate the signaling processes involved in maintenance of cellular redox homeostasis that neutralize the adverse impacts of ROS and provide NO induced defense against oxidative by improving anti-oxidant activities, carotenoid contents and electron transport under cold stress (Irshad et al., 2021).

## Melatonin Improves Photosynthetic Performance Under Cold Stress

**FIGURE 3:**
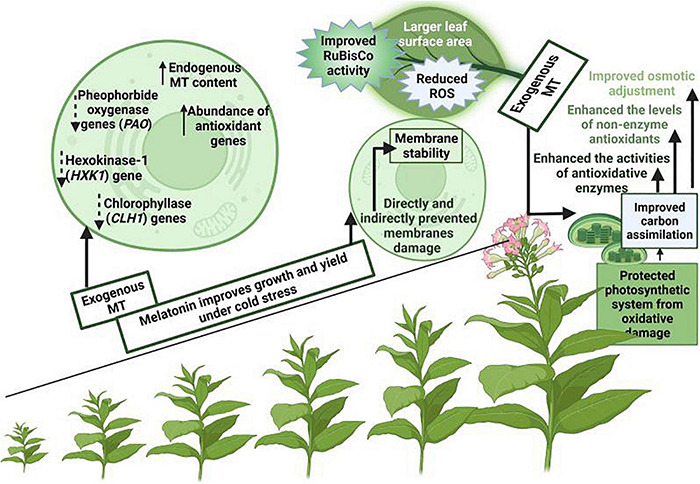
A proposed model for MT induced increase in photosynthetic under cold stress. MT supplementation protects photosynthetic apparatus, and maintains genes expression linked with chlorophyll synthesis and improves the osmotic adjustment, carbon assimilation and anti-oxidant activities and resulting in significant improvement in photosynthesis under cold stress.

Photosynthesis is an imperative physiological process that occurs in plants and it is considered as a basis of biological world, however, this process is considered to be very sensitive to cold stress (Dalal and Tripathy, 2012). Cold stress decreases the plant photosynthetic pigments, destroy chloroplasts structure, close stomata, and decreases photosynthetic rate and stomata conductance (Fan et al., 2015; Cai et al., 2016; Han et al., 2017). Melatonin supply alleviate the cold induce inhibition in plant photosynthetic efficiency, maintain lower non-photochemical quenching (NPQ) and protect the photosynthetic apparatus from cold stress (Han et al., 2017). MT application also improves the chlorophyll synthesis (Figure 3) by improving the anti-oxidant activities and protecting the photosynthetic apparatus (Irshad et al., 2021). MT supplementation improved the endogenous MT contents that decreased the expression of oxygenase (PAO) gene that is involved in chlorophyll degradation and senescence-related hexokinase-1 (HXK1) gene (Wang et al., 2013; Weeda et al., 2014). MT supply also improved the stomata conductance and improve the plant photosynthetic efficiency by increasing the carbon dioxide (CO_2_) absorption (Zhong et al., 2020).

Cold stress decreases the activities of enzymes involved in photosynthesis and RuBisCo is considered as a most important enzyme of photosynthetic process (Turk et al., 2014). Cold stress inhibited RuBisCo activity, however; MT maintained the higher RuBisCo activity and improves the photosynthetic efficiency under cold stress owing to reduced ROS production (Turk et al., 2014). The improvement in photosynthetic efficiency with MT application under cold stress is attributed to decreased ROS production, increase in light perception and RuBisCo activity (Turk et al., 2014; Erland et al., 2018; Yang et al., 2018). Additionally, MT also protects the chlorophyll degradation addition, and delays the leaf senesces which also leads to marked improvement in photosynthetic efficiency under cold stress (Han et al., 2017; Ye et al., 2020).

The exogenous application of MT also maintains the higher Fv/Fm and reduced the MDA and H_2_O_2_ accumulation which favors an increase in photosynthetic efficiency (Tan et al., 2012; Bajwa et al., 2014; Li et al., 2018a). The effect of MT on photosynthesis is concentration dependent. Since the endogenous MT varies among the species, therefore, different concentrations of exogenous MT may exert different effects on plant photosynthetic efficiency (Lazar et al., 2013). The photo-inhibition of photosystem-I (PS-I) is considered to be more dangerous as compared to PS-II, however, MT application protect the thylakoid membranes and recover the photo-inhibition of PS-1 and PS-II and maintain the higher photosynthetic efficiency under cold stress (Yang et al., 2018).

## Melatonin Improves Accumulation of Secondary Metabolites in Cold Stress

**FIGURE 4:**
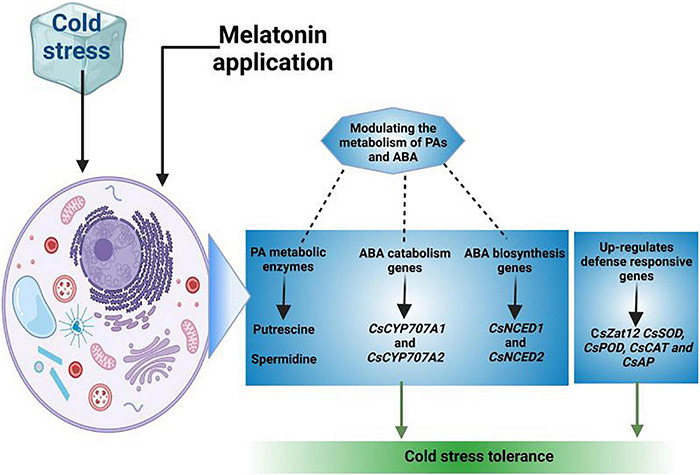
A proposed model of MT medicated polyamines accumulation for improving cold tolerance in plants. MT application upregulates genes expression linked with polyamines accumulation and genes linked with ABA synthesis and ABA catabolism. The application of MT improves polyamines accumulation and increase the genes expression to for catabolism of ABA and resulting in significant improvement in photosynthetic efficiency and cold tolerance in plants.

Phenolic compounds possess excellent anti-oxidant properties and they accumulate in plants in response to different stress conditions (Agati et al., 2007). Cold stress increased the levels of phenolic compounds while exogenous application of MT further enhanced the phenolic contents to confer the cold tolerance (Szafranska et al., 2012; Turk et al., 2014). Polyamines maintain enzymatic activities, membrane integrity and protein structures by scavenging ROS and phospholipid binding capacity (Aghdam et al., 2019). MT application improves the cold stress defense mechanism by increasing the concentration of polyamines (Figure 4) (Put, Spd, and Spm) (Cao et al., 2016). Moreover, increased expression of LeARG1 and LeARG2 encoding arginase genes, arginine decarboxylase (LeADC) and ornithine decarboxylase (LeODC) improved the chilling tolerance in plants (Zhang X. et al., 2011). Additionally, MT pre-treatments increased the accumulation of spermine, spermidine, and putrescine by regulating the S-adenosylmethionine decarboxylase (SAMDC) and tranglutaminase (TGase) activities and resulting in increase in cold tolerance (Du et al., 2021).

The increase in zinc finger protein (Zat12) gene expression involved in putrescine accumulation is also upregulated by expression of ADC1 and ADC2 genes following MT application that improved the cold tolerance in plants (Zhao et al., 2017). Moreover, MT supplementation also increased the enzymatic activities and encoding genes (CsADC and CsODC) expression level which in turn improved the polyamines accumulation and improved the cold tolerance by increasing anti-oxidant activities (Madebo et al., 2021). Gamma-aminobutyric acid (GABA) is a non-protein amino acid which is found in most of organisms (Madebo et al., 2021). The MT treatment improved the upregulation of PpGAD expression and increases ascertain of GABA in chilling stress (Cao et al., 2016). The increase in GABA accumulation following MT application serves as H_2_O_2_ scavenger which protects the membranes and improved the plant performance under cold stress (Cao et al., 2016; Wang et al., 2016).

## Melatonin Strengthens Antioxidant Defense Activities to Confer Cold Tolerance

**FIGURE 5:**
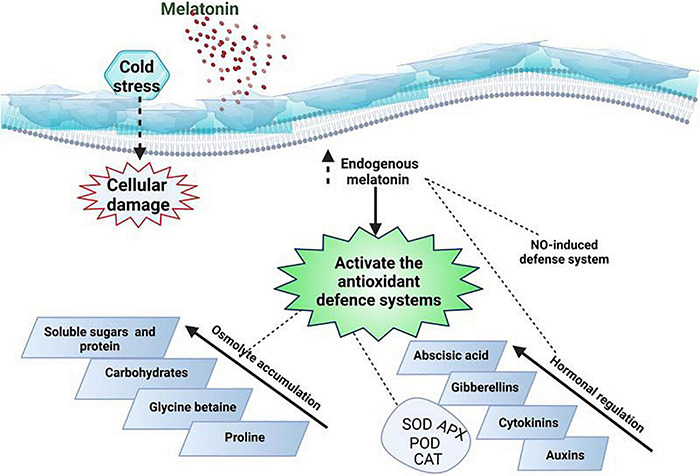
A proposed model of MT mediated increase in anti-oxidant activities and osmolytes accumulation for conferring cold stress in plants. NO: nitric acid.

Cold stress induces certain changes in plant anti-oxidant activities and these alterations are considered as mechanisms to alleviate the adverse impacts of ROS (Barand et al., 2020). MT application scavenges the ROS directly or indirectly by raising the activities of anti-oxidant (Figure 5) enzymes (Li et al., 2012; Arnao and Hernández-Ruiz, 2015a). APX, CAT and glutathione peroxidase (GPX) are considered as (Table 4) essential enzymes responsible for breaking the H_2_O_2_ into H_2_O in plant cells (Mittler et al., 2004; Aamer et al., 2018). MT application enhances the activities of aforementioned enzymes and counters the deleterious impacts of various abiotic stresses (Fan et al., 2015). MT application appreciably increased the APX, CAT, POD, and SOD (Table 4) activities which is attributed to drop in leaf temperature and increase in ROS accumulation in plant leaves (Li et al., 2018c). The reduction in ROS production by MT improved the plant performance under cold stress (Li et al., 2018c). MT seed priming and foliar application upregulated the APX and SOD activities which reduced the ROS production and conferred the cold tolerance in barley (Li et al., 2016). The exogenous MT application increased the expression of anti-oxidant genes including Cu/Zn-SOD and Fe-SOD that increased the SOD activities and improved the cold tolerance in MT treated plants (Sun et al., 2018).

**TABLE 4:** Effect of melatonin application on enzymatic and non-enzymatic activities under cold stress.

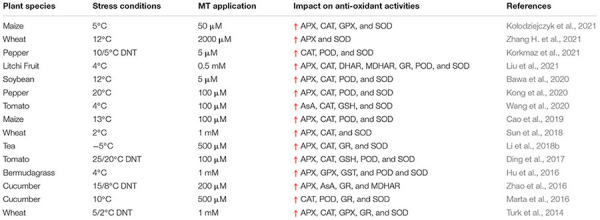

*DNT, day night temperature.*

Melatonin also induced the accumulation of anti-oxidant metabolism-related proteins and increases the potential of anti-oxidant system to scavenge the ROS under cold stress (Tan et al., 2012; Turk et al., 2014; Shi et al., 2015b). MT application also increases the stress tolerance in different plant species by inducing H_2_O_2_ as defense signaling (Shi et al., 2015b; Li et al., 2016). Plants also have to maintain optimum cellular redox homeostasis to continue normal functioning under stress conditions (Kocsy et al., 2001). Glutathione being a redox active compound maintains cellular homeostasis by affecting the different biological pathways and maintain plant performance under cold stress (Suzuki et al., 2012). MT pretreatment maintains higher GSH:GSSG ratio and reduce the ROS production under cold stress (Li et al., 2018a). Moreover, MT application also improved the AsA activity in cold stress, additionally, MT also improved GSH content by improving the activity of c-glutamylcysteine enzymes involved in glutathione (GSH) synthesis (Xu et al., 2010; Li et al., 2018a). All these findings finding indicated that MT application upregulates the activities both enzymatic and non-enzymatic anti-oxidant to confer cold tolerance in plants.

## Melatonin Increases the Expression of Stress Responsive Genes to Confer Cold Tolerance

The increase in genes expression plays an imperious role to mitigate the adverse impacts of cold stress. MT treatment appreciably improved the genes expression and improved the plant tolerance to cold stress. Likewise, MT seed treatment and foliar spray considerably increased the expression of Cu/Zn SOD, Fe/SOD gene and CAT genes which in turn improves overall plant performance and anti-oxidant activities under cold stress (Sun et al., 2018). MT markedly upregulate the expression of C-repeat-binding factors (CBFs)/drought response element binding factors (DREBs) and different cold responsive genes (COR15a and CAMTA1) and anti-oxidant genes (ZAT10 and ZAT12) that contributes to improved growth and cold tolerance in plants (Bajwa et al., 2014). MT application increased the IAA and jasmonic acid levels, however, it decreased the ABA concentration in cold stress. This indicates that MT works synergistically with IAA and jasmonic acid (JA) and anta-agonistically with ABA to regulate the plant responses to cold stress (Chang et al., 2020).

Cold stress also increased the expression of fatty acid desaturase (FAD2), conversely MT treatment lower the FAD2 expression and consequently reduced the lipid per-oxidation under cold stress (García et al., 2014; Barand et al., 2020). MT treatment upregulates stress responsive gene (CsZat12) and increases the accumulation of polyamines (Put, Spm, Spd) by altering the activity of polyamine metabolic enzymes. Moreover, MT also modulates the expression of ABA synthesis genes (CsNCED1 and CsNCED2) and ABA catabolism genes (CsCYP707A1 and CsCYP707A2) to confer cold tolerance in plants (Zhao et al., 2017). The application of MT also induces the RBOHD-dependent H_2_O_2_ generation in cold stress and increase in H_2_O_2_ promotes Ca^2+^ accumulation that sends signals for anti-oxidant activities and improve the cold tolerance (Chang T. et al., 2021). MT application also upregulate the expression of anti-oxidant genes (CsSOD, CsPOD, CsCAT, and CsAPX) that increases the anti-oxidant activities of and resultantly increased the ROS scavenging (Li et al., 2019).

## Engineering Melatonin Biosynthesis Improves Cold Tolerance

The efforts are being made to develop the transgenic plants with improved MT bio-synthesis for ensuring the cold tolerance in plants. For instance higher SNA (Serotonin *N*-acetyltransferase) specific enzyme activities were noticed in transgenic plants, and higher expression of SNA induces a significant increase in MT biosynthesis and subsequent cold tolerance (Kang et al., 2010). Likewise, over-expression of SNAT2 in rice lines increased the MT biosynthesis, which improved plant tolerance to cold stress (Hwang and Back, 2019). The *oAANAT* gene’s over-expression plays a significant role in MT biosynthesis under stress conditions. The increase in expression of the oAANAT gene enhanced the MT contents and promoted the plant growth and spike length of switchgrass under cold stress (Yuan et al., 2016). In cotton crops, over-expression of *GhM2H* gene improved the tolerance against heat and cold stress by increasing endogenous MT contents and antioxidant activities and reducing ABA accumulation (Zhang Y. et al., 2021).

The insertion of ClCOMT1 in transgenic watermelon plants significantly increased the MT bio-synthesis. ClCOMT1 expression in watermelon was also substantially increased under cold, drought, and salt stress following increased MT accumulation. Therefore, ClCOMT1 over-expression is considered a positive plant growth regulator in response to heat, cold and drought stresses (Chang J. et al., 2021). Another group of researchers identified that inserted the *ASMT* genes apple plant. They noted that ASMT genes were significantly upregulated under cold, drought and heat stress. The expression of these genes appreciably increased MT biosynthesis, which increased the plant tolerance to cold, drought, and heat stresses (Wang et al., 2022).

## Conclusion and Future Perspectives

Melatonin application effectively modulates plant growth and confers cold tolerance in plants. The exogenous MT application improved the synthesis of photosynthetic pigments and maintains membrane stability, plant water status, increasing the nutrient and water uptake, which improved plant growth under cold stress. Melatonin supplementation also alleviates the cold-induced osmotic imbalance by increasing the accumulation of different osmolytes, endogenous MT, hormones, and secondary metabolites. Moreover, exogenous MT supply also helps the cold-induced deleterious impacts by increasing the expression of different defensive genes responsible for the higher antioxidant activities under cold stress. The genes manipulation associated with enhanced MT biosynthesis also appreciably improved the cold tolerance in plants by favoring the antioxidant activities, photosynthetic performance and accumulation of different osmolytes.

Still, the role of MT in cold tolerance is not fully explored, and more research is direly needed to uncover its potential benefits under cold tolerance. The exact position of MT biosynthesis in plants requires further investigation. MT is also an unstable molecule; therefore, its transportation in plants organs under cold stress must also be studied in future research programs. The role of MT in improving root growth under cold stress is well studied; however, its role in nutrient uptake is poorly studied. Therefore, the role of MT in nutrient uptake and transportation must be explored in future research studies.

Moreover, increased endogenous MT level in plants under cold stress occurs by upregulation of MT bio-synthesis genes or MT absorption from the exogenous MT application; both mechanisms need more investigation to ensure better MT biosynthesis in plants. The role of MT on pollen viability, abscission and crop quality under cold stress must be explored at the field level. Further studies are also direly needed to identify the interaction of MT with other osmolytes and hormones under cold stress. Recent improvements in genetic engineering have provided clues to diverse complex gene-protein interactions and interconnected networks. Therefore, genetic engineering will enable us to understand better the interaction of MT with other hormones under cold stress. The role of MT in stomatal signaling under cold stress is also unknown; therefore, future research direction on this aspect would also fascinate. Plant chloroplast and mitochondria are e significant sites of ROS production. MT works as signaling molecules; therefore, it would be fascinating to explore the inter-organelle MT signaling under cold stress. Additionally, molecular mechanisms of MT in increasing the expression of antioxidant as stress-responsive genes must also be examined under cold pressure in future research studies. All these efforts will increase our understanding of the roles of MT as a potential antioxidant to be used in cold stress conditions.

## Data Availability Statement

The original contributions presented in the study are included in the article/supplementary material, further inquiries can be directed to the corresponding author.

## Author Contributions

SHQ and MUH conceived the idea and wrote the original draft. MUC, AM, MS, MaN, MuN, LB, MAA, and MA helped with organization and editing. All authors contributed to the article and approved the submitted version.

## Conflict of Interest

The authors declare that the research was conducted in the absence of any commercial or financial relationships that could be construed as a potential conflict of interest.

## Publisher’s Note

All claims expressed in this article are solely those of the authors and do not necessarily represent those of their affiliated organizations, or those of the publisher, the editors and the reviewers. Any product that may be evaluated in this article, or claim that may be made by its manufacturer, is not guaranteed or endorsed by the publisher.
